# Identifying Predictors of Inpatient Verbal Aggression in a Forensic Psychiatric Setting Using a Tree-based Modeling Approach

**DOI:** 10.1177/08862605211021972

**Published:** 2021-06-13

**Authors:** Merten Neumann, Thimna Klatt

**Affiliations:** 1 Criminological Research Institute of Lower Saxony, Hanover, Germany; 2 Criminological Research Service of the Ministry of Justice in North Rhine-Westphalia, Germany

**Keywords:** forensic psychiatry, tree-based modeling, random forest, verbal aggression, mental illness

## Abstract

Inpatient violence poses a great risk to the health and well-being of other patients and members of staff. Previous research has shown that prevalence rates of violent behavior are particularly high in forensic psychiatric settings. Thus, the reliable identification of forensic inpatients who are particularly at risk for violent behavior is an important aspect of risk management. In the present study, we analyzed clinicians’ assessments of *N* = 504 male and female inpatients of German forensic mental health institutions in order to identify risk factors for verbal institutional violence. Using a tree-based modeling approach, we found the following variables to be predictors of verbal aggression: gender, insight into the illness, number of prior admissions to psychiatric hospitals, and insight into the iniquity of the offence. A high number of prior admissions to psychiatric hospitals seems to be a risk factor for verbal aggression amongst men whereas it showed the opposite effect amongst women. Our results highlight the importance of dynamic risk factors, such as poor insight into the own illness, in the prediction of violent incidents. With regard to future research, we argue for a stronger emphasis on nonparametric models as well as on potential interaction effects of risk and protective factors.

Inpatient violence is a considerable problem in both civil and forensic psychiatric settings. Violent behavior, including verbal aggression by inpatients not only endangers the well-being of other patients and members of staff, it can also have a negative impact on the therapeutic climate. Furthermore, inpatient violence is associated with high staff turnover (Morrison et al., 2002) and causes considerable financial damage (Hillbrand et al., 1996). Hankin et al. (2011) estimated that the direct costs of inpatient aggression (excluding staff replacement and treatment costs as well as compensation claims) in psychiatric settings in the United Kingdom amount to approximately 15.2 million GBP per year (or 32.5 million in 2010 USD), which corresponds to 820 GBP (or 1,752 in 2010 USD) per incident.

Prevalence rates of inpatient violence have been found to vary greatly, ranging from 8% (Ketelsen et al., 2007) to 44% (Desmarais et al., 2010). These differences are, at least in part, attributable to differences in the definition of violence, different samples, sources of data, and follow-up periods (for reviews, see Bowers et al., 2011; Cornaggia et al., 2011). Bowers et al. (2011) found a broad range of different definitions of violence and aggression in their review of more than 120 studies. The authors noted that the vast majority of the studies used a measure of physical violence, some also included verbal violence (e.g., threats), violence against objects, sexual violence, self-harm, or combinations of these behaviors as dependent variables. Prevalence rates of violence were significantly higher when verbal violence was included in the outcome measure compared to studies that did not examine verbal aggression. The average rate of inpatient violence was also found to be significantly higher in forensic samples than in samples drawn from acute and psychiatric hospitals (Bowers et al., 2011). Methods of data collection also vary between studies, with some analyzing clinical files or incident reports (e.g., Karson & Bigelow, 1987), and others utilizing standardized, validated measures (e.g., Daffern et al., 2006; Desmarais et al., 2010), such as the Overt Aggression Scale (OAS; Silver & Yudofsky, 1987; Silver & Yudofsky, 1991). With regard to the follow-up period, it can be expected that longer timeframes are associated with a higher prevalence of aggressive behavior.

In order to develop and implement effective prevention measures, it is indispensable to know which factors increase or decrease the risk of violent inpatient behavior. Toward this end, a number of studies have been conducted to identify demographic characteristics of the patients, indicators of the patients’ criminal and institutional history, and clinical variables, including psychological disorders that predict inpatient aggression (e.g., Bowers et al., 2009; Daffern et al., 2005; Doyle & Dolan, 2006; Flannery et al., 1994; Rasmussen & Levander, 1996).

## Demographic Characteristics of the Patients

Previous research predominantly indicates that younger age is a risk factor for violence (e.g., Hoptman et al., 1999; Jeandarme et al., 2016; for a meta-analysis, see Dack et al., 2013). However, some studies have failed to find a significant association of age with violence risk (e.g., Daffern et al., 2005; Dolan et al., 2008; Fullam & Dolan, 2008; Hill et al., 1996; for a meta-analysis, see Iozzino et al., 2015). Differences between research findings could be due to the use of different statistical procedures and the inclusion of control variables. Ketelsen et al. (2007), for example, found in their study that aggressive patients were significantly younger than nonaggressive patients in a bivariate comparison. However, in a multivariate model including other risk factors, a positive, although very weak association between age and aggression was observed.

Evidence regarding the effect of gender on inpatient violence has been inconsistent. Steinert et al. (1999) who conducted a chart review of a sample of 138 patients of a psychiatric hospital in Germany with a first episode of schizophrenia or schizoaffective disorder reported that male patients were more likely to become aggressive than their female counterparts. On the other hand, Chan and Chow (2014) found female gender to be a risk factor for aggression in their sample of forensic inpatients in Hong Kong. The same was found by Serper et al. (2005) in a sample of acute inpatients in New York City. These contradicting results might be due to different settings or samples. Two meta-analyses showed that among forensic samples, males were less likely to be aggressive than females, whereas the opposite was true for patients in acute wards (Bowers et al., 2011; Dack et al., 2013). Therefore, there could be an interaction effect of gender and setting (forensic vs. civil) underlying the different results. Inconsistencies between research findings could also be due to the use of different outcome measures. Daffern et al. (2005), who investigated aggressive incidents in a forensic psychiatric hospital, found that males and females were equally likely to be violent, but females were more likely than males to be repeatedly involved in aggressive incidents.

## Criminal and Institutional History

A particularly prominent predictor of aggressive behavior is previous aggression (e.g., Flannery et al., 1994; Iozzino et al., 2015; Karson & Bigelow, 1987). A meta-analysis by Dack et al. (2013) indicates that a history of violent behavior and convictions for violent crimes are both associated with an increased risk of inpatient aggression. However, in a sample of forensic inpatients with schizophrenia, no significant differences were found between aggressive and nonaggressive patients with regard to their history of violence (Fullam & Dolan, 2008). Heilbrun et al. (1998), who studied a sample of forensic inpatients in Florida also reported that previous acts of violence were not associated with aggression when controlling for the patients’ psychopathy score. Consequently, the relationship between present and future violence might not be as clear-cut in special inpatient populations.

Some studies conducted in forensic psychiatric settings have included measures of past criminal behavior in general, not limited to violence. Daffern et al. (2005) compared the severity of the index offence of aggressive and nonaggressive patients in an Australian forensic hospital and did not find significant differences. Investigating a sample of male forensic inpatients with schizophrenia, Fullam and Dolan (2008) found only nonsignificant differences between violent and nonviolent inpatients with regard to the number of their previous offences. Results from a study by Hoptman et al. (1999) also showed that the number of arrests for violent offences and for nonviolent offences were similar between forensic inpatients who were assaultive and those who were not assaultive during hospitalization. However, both violent and nonviolent offences were positively associated with the number of assaults during hospitalization. This shows that the relevance of certain variables in predicting inpatient aggression can depend very much on the type of outcome measure employed.

In addition to the patients’ criminal history, researchers have also investigated the potential relation between previous admissions to psychiatric hospitals and inpatient violence (for a meta-analysis, see Dack et al., 2013). Mellesdal (2003), for example, found that the mean number of previous admissions was significantly higher among aggressive patients than among nonaggressive patients in her study of verbal and physical violence on a Norwegian acute inpatient ward. The number of previous admissions was also found to be positively associated with aggressive behavior in a sample of more than 2,000 patients in a German psychiatric hospital (Ketelsen et al., 2007). The effect remained significant even after controlling for other risk factors, including current age, involuntary admission, and being diagnosed with schizophrenia.

## Clinical Variables

With regard to the potential effect of different disorders on the proneness to engage in inpatient violence, previous research has produced mixed results. Schizophrenia has received particular attention as a potential predictor of inpatient violence. Schizophrenic patients have been reported to be more likely to engage in violence than patients with other diagnoses in several studies (e.g., Karson & Bigelow, 1987; Ketelsen et al., 2007; Shah et al., 1991). Hoptman et al. (1999) did not find schizophrenia to be significantly related to violence risk. However, a dual diagnosis of schizophrenia and substance abuse or dependence was positively related to violent behavior. Two meta-analyses showed contradictory results with regard to the effect of schizophrenia on inpatient aggression: Dack et al. (2013) found that schizophrenic patients had a heightened risk of being aggressive, whereas Iozzino et al. (2015) did not find a significant association between schizophrenia and violent behavior. The reason for these contrary findings could be that Iozzino et al. (2015) used aggregate level ward characteristics (e.g., the proportion of patients with schizophrenia) as predictors. Dack et al. (2013), on the other hand, analyzed individual inpatient factors.

Personality disorders have also been studied as a potential predictor of inpatient aggression. Research conducted in Belgium, Hong Kong, the United Kingdom, and the United States indicates that personality disorders are indeed a risk factor for inpatient aggression (e.g., Chan & Chow, 2014; Hillbrand et al., 1996; Jeandarme et al., 2016; Soliman & Reza, 2001). However, other studies conducted in both forensic and civil psychiatric settings did not find a significant association between personality disorder and violence risk (e.g., Dolan et al., 2008; Ketelsen et al., 2007; for a meta-analysis, see Iozzino et al., 2015). These inconsistencies between studies could be due to variations in the outcome variable. Jeandarme et al. (2016) conducted a number of logistic regression analyses and found that a diagnosis of personality disorder was significantly associated with verbal aggression, but not with physical violence nor with a combined measure of physical and verbal violence.

Substance use and related disorders have also been investigated as potential risk factors for inpatient aggression. Several studies (e.g., Chan & Chow, 2014; Dack et al., 2013; Flannery et al., 1994; Hill et al., 1996) showed a higher risk of engaging in aggressive behavior in patients with a history of substance abuse. Dack et al. (2013) found in their meta-analysis that past substance misuse also heightens the risk of being repeatedly aggressive (vs. being a one-time offender) while hospitalized. Daffern et al. (2005) reported that the average number of substances used in the previous year was significantly higher among aggressive patients than among nonaggressive patients within a forensic psychiatric hospital. However, the two groups did not differ significantly with regard to their lifetime history of substance use. Some studies failed to find any effect of substance abuse on inpatient violence (e.g., Fullam & Dolan, 2008; Hoptman et al., 1999; Steinert et al., 1999). These different findings might be due to differences in the temporal proximity of substance use and violent behavior, as well as to variations in the predictor and outcome variables. Jeandarme et al. (2016), for example, found a significant bivariate association between alcohol use and the severity of violence against others. Illicit drug use, on the other hand, did not have an effect on inpatient violence. Bowers et al. (2009) found in their study of 136 acute psychiatric wards in England that substance abuse was associated with verbal aggression, but not with physical aggression against people or objects.

Some research on correlates of inpatient aggression has focused on characteristics of the current hospitalization in general and the patients’ behavior as well as indicators of treatment process in particular. The length of stay of the index hospitalization was found to be uncorrelated with verbal and physical aggression in both forensic and civil inpatient samples (e.g., Doyle & Dolan, 2006; Hillbrand et al., 1996; Jeandarme et al., 2016; Karson & Bigelow, 1987; Shah et al., 1991).

With regard to the compliance with treatment, Hillbrand et al. (1996) found in their study on staff injuries in a maximum-security forensic hospital that nonadherence to medication regimes and to psychosocial treatment was associated with institutional violence. A study of inpatients in three medium-security units in Flanders showed a positive and statistically significant association between verbal violence and noncompliance (i.e., not adhering to treatment rules; Jeandarme et al., 2016). Absconding from the unit, the premises, or during an (un)supervised leave was positively related to physical violence against other people. Both effects remained significant after controlling for other risk factors (Jeandarme et al., 2016). Hill et al. (1996) also reported significant correlations between noncompliance and aggression, as well as between escape and aggression in a sample of male offenders in a Texan maximum-security forensic psychiatry. Based on their study of forensic inpatients in the United Kingdom, Doyle and Dolan (2006) noted that a compliant interpersonal style seems to be a protective factor against verbal and physical violence.

Some previous research has investigated the patients’ insight into their illness and risk as potential correlates of violent behavior. Studying a sample of psychotic male forensic inpatients in New York City, Alia-Klein et al. (2007) reported that insight into illness was significantly related to the severity of violence, even after controlling for a number of other predictors, including age, substance use, and medication adherence. In another study examining male patients in a secure psychiatric facility in England, it was found that a lack of insight was among the strongest predictors of inpatient violence (Grevatt et al., 2004).

Overall, previous research findings regarding the effect of clinical variables on inpatient aggression indicate that schizophrenia and substance abuse increase the risk for inpatient aggression. Additionally, compliance with treatment and insight into the illness and risk have been found to be strong protective factors. Evidence regarding the association of variables relating to demographic characteristics of the patients and their institutional and criminal history is generally less clear, with the exception of the effects of age, violent behavior in the past, and previous admissions.

Inconsistencies between the results of different studies are probably related to the utilization of different samples, different data collection methods, different predictors and outcome measures, and different statistical procedures. Most of the previous studies on risk factors for inpatient aggression have either conducted bivariate comparisons of violent and nonviolent patients or employed a main-effects regression approach. Both methods do not allow for the identification of different risk factors for different subgroups of patients. However, variation between study findings could be due the fact that some risk factors for violent behavior only have an effect among certain subgroups of patients (Hodgins et al., 2003; Steadman et al., 2000). Substance use disorders, for example, have been found to increase the risk for violent behavior in people with schizophrenia, but not among schizophrenics with psychopathic traits (Tengström et al., 2000, 2004).

## The Present Study

Most research on risk factors associated with institutional violence by psychiatric patients has focused on physical aggression in civil psychiatric settings (for a meta-analysis, see Iozzino et al., 2015). Studies examining verbal aggression in forensic psychiatric hospitals and wards are comparably scarce. Therefore, and due to the fact that rates of inpatient aggression are particularly high in forensic samples (Bowers et al., 2011), studying the risk factors associated with inpatient verbal violence in forensic psychiatric settings is of considerable importance.

The aim of the present study is thus to further investigate risk factors for institutional aggression within a forensic psychiatric setting based on previously established risk factors. Furthermore, the predictive performance of the resulting model will be evaluated using a confusion matrix. The contradictory results of previous studies could be related to different samples (e.g., in terms of psychiatric diagnoses), different settings (e.g., civil vs. forensic, different countries), and data collection methods. Additionally, potentially complicated interaction effects or unclear (e.g., nonlinear) associations between predictors and dependent variables could conceal the underlying effects. Therefore, standard predictive methods alone, such as linear regression, are not advisable. An appropriate alternative for classification and prediction purposes are tree-based models (Fritsch et al., 2019), which were employed in the present study.

## Method

### Sample and Data

Within the German forensic mental health system, patients can be admitted to a forensic psychiatry on the basis of section 63 and section 64 of the German criminal code. Patients confined based on section 63 committed an offence while suffering from a severe mental disorder (e.g., schizophrenia, personality disorder, paraphilia) or an extreme mental state (e.g., extreme emotional distress) which resulted in them having no or only diminished criminal responsibility for the crime. Patients who are admitted on the basis of section 63 of the German criminal code are incarcerated for an indefinite amount of time. Their release prospects are mainly determined by their risk assessment (Edworthy et al., 2016; Müller-Isberner et al., 2000).

Patients admitted to a forensic psychiatry based on section 64 of the German criminal code have shown problems with substance abuse that are linked to their criminal behavior. Furthermore, to be admitted to a forensic psychiatry based on section 64, some prospect of treatment success is required. Diminished criminal responsibility is not a prerequisite and the duration of treatment in the institution is usually limited to two years (Edworthy et al., 2016). Some German forensic mental health institutions only treat patients admitted based on section 63 (so-called forensic hospitals), others specialize on treating patients admitted based on section 64 (so-called forensic detoxification clinics), and some accept both.

The present study uses data from a research project conducted by the Criminological Research Institute of Lower Saxony. The aim of the research project was to evaluate the gradual release process in all forensic mental health institutions (*N* = 10) in Lower Saxony, a federal state in northern Germany (Neumann et al., 2019; funded by the Ministry of Social Affairs of Lower Saxony).

Data were gathered from all patients (confined based on section 63 or section 64 of the German criminal code) who underwent an external risk assessment in relation to their application for unsupervised leave. The sample comprises a total of 668 patients who were granted temporary unsupervised leave between 2006 and 2016 (average overall number of occupied beds per year: 1265). For the risk assessment, the patients’ main therapists completed a questionnaire devised by the forensic mental health institutions in Lower Saxony containing demographic variables as well as static and dynamic risk and protective factors (no validated instruments are included; the questionnaire can be found in Neumann et al., 2019). This questionnaire is then handed to external clinicians (3 people from different clinics) together with the patients’ file for an external expert assessment regarding the question of unsupervised leave. When a patient was granted unsupervised leave from the institution, any rule breaking behavior, including verbal aggression, was recorded for the following 12 months. Both sources of information (questionnaires completed by main therapists and data on verbal aggression) were integrated into one data set.

Amongst the 668 patients in our sample were 164 with missing data regarding verbally aggressive behavior (mostly because the necessary timeframe of one year since the risk assessment had not been reached at the time of data collection). These cases were excluded, leaving 504 patients for the analysis. Missing values on all other variables were imputed using *predictive mean matching* (Little, 1988) for numeric variables and logistic regression (White et al., 2010) for binary variables. All imputations were conducted using the package *mice* (Van Buuren & Groothuis-Oudshoorn, 2011) for the statistics software R (R Core Team, 2020). For all variables included in the imputation process the proportion of missing values was under 4%.

### Measures

#### Dependent variable.

Six months and 12 months after the patients were granted permission to leave the institution unsupervised, their main therapists completed a standardized form including questions about any rule breaking behavior during the preceding six months. The information for this assessment was mainly gathered from the patients’ files. The item of interest for the present study is *severe verbal aggression* (e.g., harsh insults, threats) against other patients or members of staff. The indicator for verbal aggression was collapsed across both times of measurement to form a binary indicator of verbally violent behavior within a timeframe of 12 months.

#### Predictor variables.

See Table 1 for an overview of the predictor variables used in this analysis.

**Table 1.** List of Predictor Variables Used for Tree-based Modeling.

**Institutional history**	**Clinical variables^o2^**	**Psychological disorders^d^**	**Offences^d^**	**Control variables**
Number of prior imprisonments^o1^	Therapeutic contact	Psychotic disorder	Property offence	Basis of confinement^d^
Number of prior admissions to psychiatric hospitals^o1^	Cooperation during treatment	Disorder of sexual preference	Homicide	Gender^d^
Prior admission to a forensic hospital^d^	Motivation regarding treatment	Personality disorder	Sex offence	Age^m^
Prior admission to a forensic detoxification clinic^d^	Insight into illness	Substance use disorder	Violent crime	
Abuse of short leave^d^	Insight into the iniquity of the offence	Mental retardation	Arson	
Length of stay^m^	Therapeutic progress			
	Suicide risk			

*Note.* ^d^ = dichotomous, ^o1^ = 5-point ordinal scale (0, 1-2, 3-5, 6-10, >10), ^o2^ = 5-point ordinal scale (very low-very high), ^m^ = metric.

#### Statistical Analysis

The present study utilizes a nonparametric approach called tree-based modeling. Tree-based models (also called recursive partitioning or decision trees) try to identify the most distinct groups of cases within a data set with regards to the dependent variable. Most of the implementations of these kinds of models use algorithms which follow a so-called greedy top-down approach. This means that the algorithm starts by investigating every possible binary split amongst the predictor variables and chooses the one split that leads to the most substantial difference between the two resulting groups regarding their distributions on the dependent variable (generally measured by impurity or entropy measures such as the Gini-index; e.g., James et al., 2017; Strobl et al., 2009). Over the past 50 years, numerous algorithms have been developed within the framework of tree-based modeling (Loh, 2014), and especially in recent years the popularity of this method has grown substantially amongst researchers from various fields (e.g., Fritsch et al., 2019; Kern et al., 2019; Mößle et al., 2017; Schivinski, 2021; Neumann et al., 2019). The major advantages of tree-based models include the ability to handle data sets with a large number of predictor variables (“large *p*, small *n* problem”), the possibility to detect intricate interaction effects, and the intuitive visualization method as a tree-structure. The major disadvantage of tree-based models lies in their instability (Strobl et al., 2009). Even the smallest changes in the learning data set can lead to completely different models as every split is dependent on the splits that come before. To compute more stable models, one can turn to random forests (e.g., James et al., 2017; Strobl et al., 2009). A random forest consists of multiple single tree-models, each computed on a random subset of cases and predictor variables of the original data set. These different trees all contribute to the final predictions in some form of “democratic voting process”. This procedure counters problems with overfitting and leads to more stable predictive models.

We first computed a single tree model to visualize groups of differential risk for verbally aggressive behavior. Specifically, we used *conditional inference trees* (Hothorn et al., 2015) implemented in the R (R Core Team, 2020) *partykit* package (Hothorn & Zeileis, 2015). This specific method employs *p*-values as the splitting criterion (Hothorn et al., 2006).

Furthermore, we computed a random forest to generate stable predictions based on our predictor variables. Here, we used *conditional inference forests* (Hothorn et al., 2006), also implemented in the *partykit* package (Hothorn & Zeileis, 2015) to compute predicted probabilities of violent behavior for every patient in our sample. To counter possible issues associated with overfitting, we computed predictions on the so-called *out-of-bag sample* (Breiman, 1996). Using the predicted probabilities of our model, we performed an ROC analysis (Fawcett, 2006) as implemented in the *pROC* package (Robin et al., 2011) to evaluate model performance based on the AUC, and to dichotomize our predictions at the best possible split-probability with regards to sensitivity and specificity (Youden, 1950). Furthermore, conditional variable importance measures were used to identify the predictors with the highest impact on predictive performance within the forest model (Hapfelmeier et al., 2014; Strobl et al., 2008). Unfortunately, the variable importance measure gives no indication about the nature of the relationship between predictor and criterion (i.e., direction and size of the effect). We therefore computed additional logistic regression models to at least gain insight into the effects of the predictor variables with the highest variable importance measures on the outcome measure.

## Results

### Descriptive Statistics and Single Tree Model

Patients ranged in age from 19 to 79 years (*M* = 39.1, *SD* = 11.5) and the vast majority of them were male (92.5%). The most common types of psychiatric diagnoses in this sample were substance use disorders (F1X.XX, 69.2%), personality disorders (F6X.XX without F65.XX, 48.4%), and schizophrenic disorders (F2X.XX, 24.2%). 69.0% of the patients had committed a violent crime (e.g., assault, robbery) in their past, 36.3% a homicide, and 40.7% a sex offence. The comparably high rate of sex offenders among the forensic inpatients is most likely a consequence of the admission practice in Germany.

In the full sample, 58 (11.5%) of the 504 patients showed verbal aggression in the institution during the period of observation. 27.6% of the patients who were verbally aggressive also engaged in physical violence. Only four patients showed physical aggression but no verbal aggression. Thus, no further analyses regarding physical aggression were conducted.

**Figure 1.**
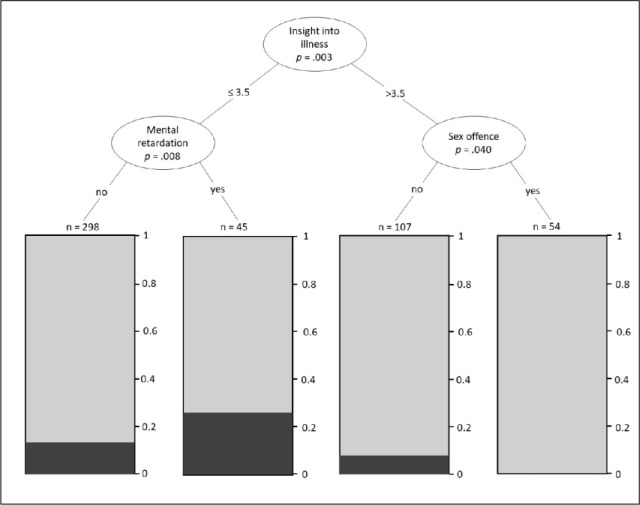
Tree-based model with the following specifications: α = .05 (no correction); maximum depth = 3; minimum group size = 25. The bars represent the prevalence of verbal aggression in each subgroup.

Figure 1 shows the tree-model. The first split identified by the model is related to the patients’ insight into their own illness. 14.6% of patients with low to medium values on this variable showed verbally aggressive behavior, whereas only 5.0% of patients with a high insight into their own illness were registered as becoming aggressive within the institution. Amongst the patients with low or medium insight into their own illness, one can identify a group of patients with a relatively high rate of verbal aggression (26.7%), which is further characterized by a diagnosis of mental retardation (F7X.XX). Patients with a high insight into their illness and a registered sexual offence showed no verbal aggression during the observation period.

### Forest Model

**Figure 2.**
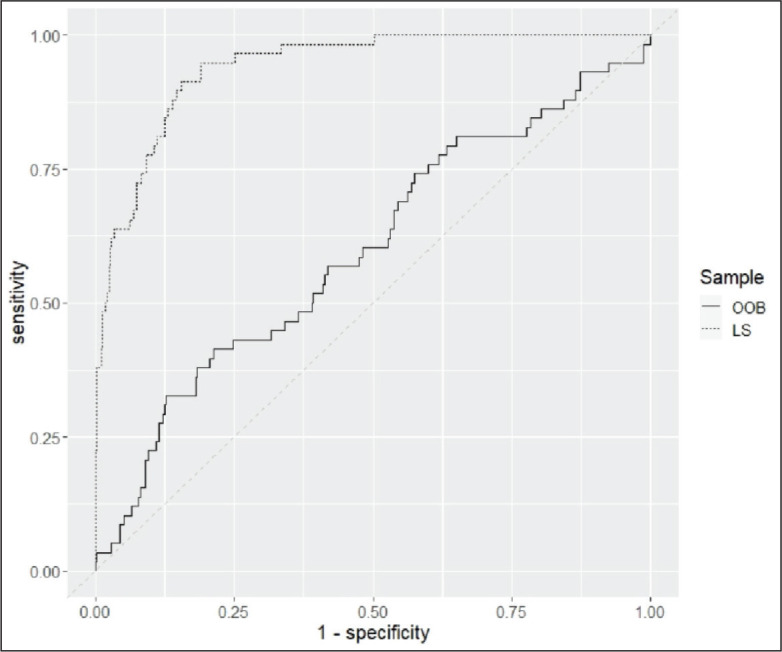
ROC-curves for predictions based on the learning sample (LS) and the out-of-bag sample (OOB). Specifications for the forest model: number of trees = 15.000; α = .3 (no correction); variables per tree = 7; weights are based on the base rate of verbal aggression.

Figure 2 shows the ROC curves resulting from the predicted probabilities for verbal aggression based on the forest model. One curve depicts the ROC curve for predictions on the learning sample (LS) and the other curve shows the predictions for the out-of-bag sample (OOB).

Both ROC curves reveal significant AUCs (LS: .94; 95% CI .91, .997; OOB: .60; 95% CI .52, .68), but the AUC value for the predicted probabilities of the LS is significantly higher than the respective value for the OOB (*Z* = 10.3, *p* < .001). As this difference is likely due to overfitting, only the OOB-predictions are considered for further analysis. Using Youden’s *J* statistic (Youden, 1950), the best cut-off probability was identified at .23, and the predicted probabilities of the forest model were dichotomized accordingly.

**Table 2.** Confusion Matrix for OOB-predictions.

		**Prediction of verbal aggression**	
		no	yes
**Verbal aggression**	no	**351**	95
	yes	34	**24**

*Note.* Bold numbers show correct predictions.

Table 2 shows the confusion matrix for the dichotomized predictions based on the OOB. The overall hit rate is .74, but as both the base rate (.12) and the selection rate (.24) are fairly low, the hit rate as a measure of predictive performance is flawed. The relative improvement over chance takes these factors into account and yields a value of .91 (see Farrington & Loeber, 1989). The model shows a specificity of .79 and a sensitivity of .41. It thus seems that the negative predictions are mostly accurate (*Negative Predictive Power* = .91), whereas the positive predictions contain many false positives (*Positive Predictive Power* = .20).

### Variable Importance Measures

**Figure 3.**
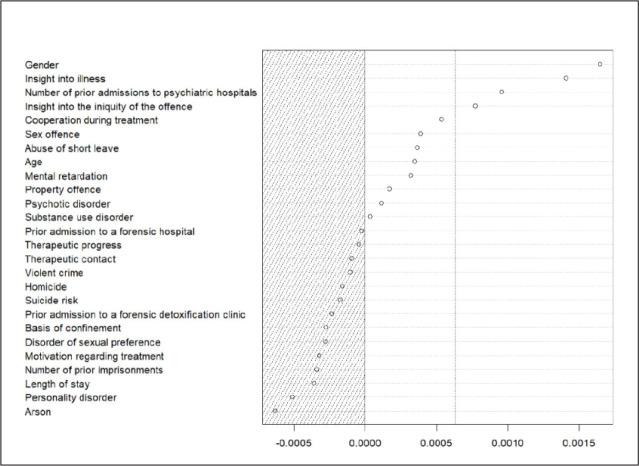
Conditional variable importance measures (threshold = .8) based on the OOB sample. The shaded area contains variable importance values below 0. The dashed vertical line represents a cut-off criterion for relevant predictor variables.

Figure 3 displays the predictor variables that were integrated into the forest model and their corresponding variable importance measures. The vertical dashed line represents the absolute value of the lowest negative variable importance measure and can be interpreted as a conservative selection criterion for relevant predictor variables (Strobl et al., 2009).

Using this analysis, four predictor variables with a meaningful impact on the predictive performance of the forest model were identified: *gender, insight into illness, number of prior admissions to psychiatric hospitals*, and *insight into the iniquity of the offence*.

### Logistic Regression

Table 3 displays the bivariate logistic regression models that were computed using the predictor variables identified via the variable importance measures. In addition, nonparametric correlation coefficients (rank-based) are reported to account for any association that is not strictly linear but still monotone.

**Table 3.** Bivariate Logistic Regression Models for the Prediction of Verbal Aggression and the Nonparametric Correlation Coefficient G.

	*B*(SE)	*p*	Exp(*B*)[95% CI]	Pseudo *R*²*	*G*^†^[95% CI]
Insight into the iniquity of the offence	−0.262(0.124)	**.035**	0.770[0.605, 0.986]	.017	−.215[−.405, −.025]
Number of prior admissions to psychiatric hospitals	0.136(0.109)	.215	1.145[0.918, 1.412]	.006	.124[−.085, .334]
Insight into illness	−0.425(0.146)	**.004**	0.654[0.488, 0.868]	.034	−.293[−.476, −.109]
Gender	−0.608(0.444)	.171	0.544[0.240, 1.401]	.007	−.295[−.692, .102]

*Note.* Bold numbers show significant *p*-values (α = .05); *Nagelkerke *R*² (Cragg & Uhler, 1970; Nagelkerke, 1991); ^†^Goodmann-Kruskal’s Gamma (Goodmann & Kruskal, 1954).

The bivariate models and the correlation coefficients revealed significant main effects of the variables: *insight into the iniquity of the offence* and *insight into illness*. Higher values on both variables were associated with a lower risk for verbal aggression. The remaining variables showed no significant main effect on verbal aggression.

*Gender* did not show any significant main effect even though it constitutes the predictor variable with the highest variable importance measure. To further clarify these findings a multivariate logistic regression model was computed (see Table 4).

**Table 4.** Multivariate Logistic Regression Model for the Prediction of Verbal Aggression.

	*B* (*SE*)	*p*	Exp(*B*)	[95% CI]
Insight into the iniquity of the offence	−0.683 (0.506)	.177	1.981	[0.797, 6.238]
Number of prior admissions to psychiatric hospitals	−1.099 (0.565)	.052	0.333	[0.081, 0.839]
Insight into illness	−1.666 (0.712)	**.019**	0.189	[0.034, 0.625]
Gender (male)	−4.499 (2.361)	.057	0.011	[0.000, 0.866]
Gender (male) *Insight into the iniquity of the offence	-0.836 (0.529)	.114	0.434	[0.133, 1.135]
Gender (male) * Number of prior admissions to psychiatric hospitals	1.297 (0.578)	**.025**	3.657	[1.404, 15.313]
Gender (male) * Insight into illness	1.430 (0.734)	.052	4.177	[1.194, 24.169]

*Note.* Bold numbers show significant *p*-values (α = .05); Nagelkerkes R² (Cragg & Uhler, 1970; Nagelkerke, 1991): .091.

**Figure 4.**
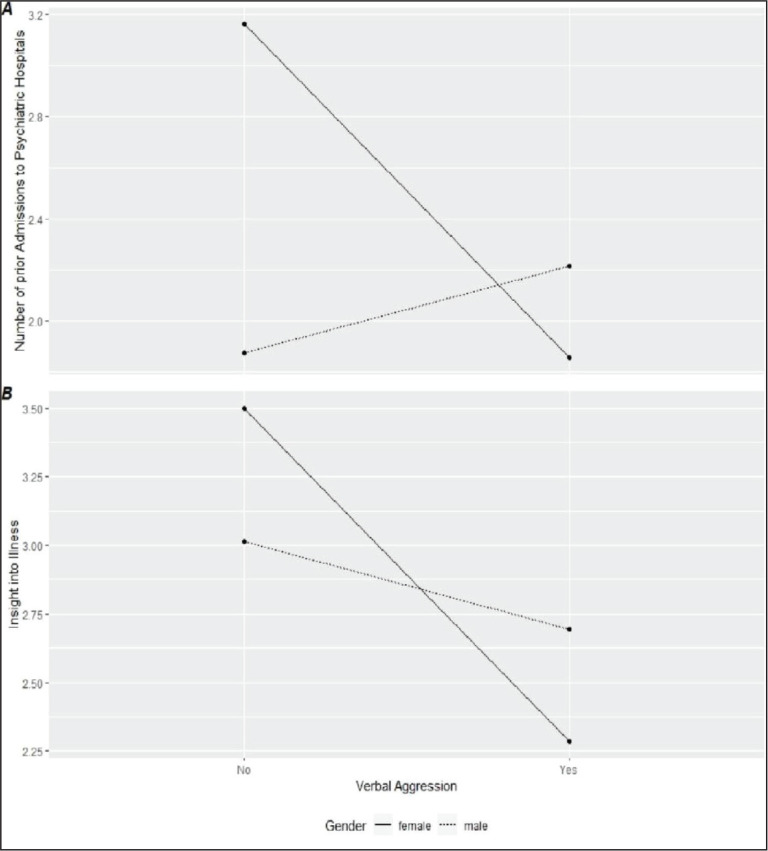
Interactions between number of prior admissions to psychiatric hospitals and gender (A), and insight into illness and gender (B).

Within the regression model, two notable interaction effects emerge (see Figure 4): *gender* x *insight into illness* (*OR* = 4.18; *p* = .05) and *gender* x *number of prior admissions to psychiatric hospitals* (*OR* = 3.66; *p* = 0.03). In our sample, the negative effect of the insight into one’s illness on verbal aggression is stronger for women than it is for men. In regard to the number of prior hospital admissions, the direction of the effect changes depending on the gender (m: positive; f: negative).

## Discussion

The present study aimed to further investigate predictor variables for inpatient verbal aggression in a forensic psychiatric setting. As previous studies relied mostly on linear regression models or bivariate analyses, we chose to include a nonparametric machine-learning approach in our analysis. Using tree-based modeling, we were able to take into account nonlinear relationships and intricate interaction effects which cannot be detected using analyses like multiple regression.

When interpreting the results of this study, some limitations need to be noted. Firstly, our sample consisted of a subsample of inpatients in German forensic mental health institutions, namely those who were granted temporary unsupervised leave. Therefore, the sample is not representative of the entire population of forensic inpatients. It is fair to assume that the patients in our sample were more advanced in their treatment process than a sample of randomly selected patients. This could also explain why the prevalence rates found in our study are relatively low compared to other research (e.g., Desmarais et al., 2010).

Furthermore, verbally aggressive incidents were assessed by therapists using a period-based (six months) standardized form. This form is not comparable to (mostly incident-based) validated report forms (e.g., OAS; Silver & Yudofsky, 1987, 1991) and does not allow further analysis of different forms of aggressive behavior and context factors of single incidents of aggression (for an overview of different types of measurement, see Nijman et al., 2006).

Despite the fact that our study shares some limitations with much of the previous literature (primarily regarding sampling and assessment of aggression), it provides some valuable insights into the predictors of verbally violent behavior in a forensic psychiatric setting. Our results suggest that patients who do not see their illness as a problem are at a particularly high risk for verbal aggression. This is supported by the variable importance measures of the random forest suggesting that dynamic clinical variables regarding the patients’ insight into the problems associated with their illness and their criminal behavior play a major role in predicting inpatient verbal aggression. This finding is in line with previous research showing that a lack of insight constitutes a risk factor for violent behavior (Alia-Klein et al., 2007; Grevatt et al., 2004).

The variable importance measures furthermore evince the predictive importance of gender and the number of prior admissions to psychiatric hospitals. Further analyses using bivariate logistic regression models and nonparametric correlation coefficients did not show any significant main effects of these predictors. This indicates that these variables are only important in interaction with other predictors. A subsequent multivariate logistic regression analysis revealed a significant interaction effect between gender and the number of prior admissions to psychiatric hospitals. A high number of prior admissions seems to be a risk factor for verbal aggression amongst men, whereas it showed the opposite effect amongst women. It is possible that these differential effects emerge because of differing diagnoses within the two subgroups. For example, men were more likely to exhibit a substance use disorder (m: 70.6%; f: 52.6%) than women, and women were more likely to show a psychotic disorder (m: 21.2%; f: 60.5%) than men within our sample. To further analyze this interaction, a sample including more women would be necessary.

A number of variables that have been found to be predictive of inpatient violence in previous research (e.g., a history of violence, personality disorders, (non)compliance with treatment; Chan & Chow, 2014; Dack et al., 2013; Hill et al., 1996; Hillbrand et al., 1996; Jeandarme et al., 2016) have produced nonsignificant results in our study. The differences between samples and outcome variables might account for these differences. The legal contexts and forensic mental health systems in different countries influence which patients enter the system in the first place. The treatment approach, staffing, and accommodation are also likely to differ internationally. Furthermore, we used verbal aggression only as our dependent variable, whereas most previous research focused on physical aggression (e.g., Fullam & Dolan, 2008; Rasmussen & Levander, 1996) or a combined measure of verbal and physical aggression as outcome measures (e.g., Daffern et al., 2006).

Overall, the most striking results of our analysis are the predictive importance of dynamic factors regarding the understanding of the problems related to the patients’ mental illness and criminal behavior as well as the moderating role of the patients’ gender. Nevertheless, it should be noted that the overall predictive performance of the forest model was low. Although the forest model showed a significant AUC and performed reasonably well regarding the hit rate, a further evaluation of model performance measures revealed unsatisfactory results in terms of sensitivity and positive predictive power. An improvement of sensitivity seems only possible with a strong increase in false positive prognoses. This means that the predictor variables that were considered in this model are not specific enough to reliably identify individual forensic patients who exhibit verbal aggression during the 12 months following assessment. Nevertheless, a significant advantage of predictive performance over chance suggests that the model can identify groups of patients with an elevated risk for verbally aggressive behavior.

### Directions for Future Research

The results of our study indicate some directions for future research. The low positive predictive power found in our analyses shows that important information is missing to make specific predictions with regard to verbally aggressive inpatient behavior. One reason might be that more proximal factors are needed to predict aggressive incidents within forensic mental health institutions. Chan and Chow (2014) analyzed daily risk assessments using short-term risk assessment tools (e.g., Brøset Violence Checklist [BVC]; Almvik et al., 2000; Dynamic Appraisal of Situational Aggression: Inpatient Version [DASA: IV], Ogloff & Daffern, 2006) and found promising results. Schuringa et al. (2018) used a monitoring tool for treatment progress and dynamic risk indicators (Instrument for Forensic Treatment Evaluation [IFTE]; Schuringa et al., 2014) to predict violent behavior (physical or verbal) amongst patients in a forensic psychiatry in the Netherlands. Their study shows that the *Problematic Behavior* dimension of the IFTE significantly predicts violent behavior in the six months after an IFTE-measurement. Therefore, it might be advantageous to further investigate continuous risk monitoring and management using standardized measurement tools within forensic psychiatric settings to predict violent incidents.

Our study also suggests that more research is needed that examines potential interaction effects. Differences between bivariate logistic regression models and tree-based models indicate that some variables are only of importance in combination with other variables. Larger sample sizes are needed to further investigate these possible interactions (e.g., the interaction of gender and problem insight). In addition, future research might benefit from tree-based models as an alternative to linear regression models. Our study, amongst others (Fritsch et al., 2019; Kern et al., 2019) demonstrates the usefulness of nonparametric models in explorative research. The assumption of a particular kind of statistical relationship (e.g., linear) needs a theoretical or empirical foundation. A disregard of the underlying statistical association between variables might produce seriously misleading results (e.g., Anscombe, 1973). Therefore, if no assumptions about the association between the predictors and the criterion can be made, using linear regression as the default statistical analysis can be highly problematic. Especially the combination of tree-based modeling and linear regression analysis might prove valuable in future research endeavors.

## Author Biographies

**Merten Neumann**, Dipl.-Psych., is a research associate at the Criminological Research Institute of Lower Saxony (KFN). His research focusses on different aspects of the correctional system with a special emphasis on risk prediction procedures in forensic mental health institutions.

**Thimna Klatt**, Dr., Dipl.-Psych., M.Sc., is a research associate at the Criminological Research Service of the Ministry of Justice in North Rhine-Westphalia, Germany. Her research focusses on corrections and short-term detention for young offenders. She is particularly interested in predictors of violent and drug misconduct within correctional institutions.
